# Work Experiences Among Infection Control Link Nurses in ICUs: A Qualitative Study

**DOI:** 10.1155/jonm/7671380

**Published:** 2025-08-18

**Authors:** Huiling Pan, Chuanlai Zhang, Ruiqi Yang, Yongjuan Chen, Zonghong Zhang

**Affiliations:** ^1^Intensive Care Unit, The Second Affiliated Hospital of Chongqing Medical University, Yuzhong, Chongqing, China; ^2^School of Nursing, Chongqing Medical University, Yuzhong, Chongqing, China

**Keywords:** cross-infection, infection control, intensive care unit, nurses, qualitative research

## Abstract

**Background:** ICU infection control link nurses play a vital role in preventing healthcare-associated infections. However, a few studies have focused on their experiences, problems, and additional needs in this role.

**Objectives:** To explore the work experience of ICU infection control link nurses.

**Methods:** This study employed a qualitative research approach using descriptive phenomenology. Between July and September 2024, 12 ICU infection control link nurses were recruited from 12 hospitals in Chongqing, China, through purposive sampling with maximum variation. Semistructured interviews were conducted with these participants. Interview data were analyzed using the Colaizzi method.

**Results:** The work experiences of ICU infection control link nurses can be summarized into 3 themes and 11 subthemes: role cognition and growth (role perception, role identity, and enhancement of personal abilities); perceptions of clinical practice (questioning and affirmation of professional recognition, emergence and resolution of burnout, teamwork issues and improvement measures, insufficient priority of quality control, and unsolvable problems); and work demands (systematic training, hospital support, and qualification recognition and career development).

**Conclusions:** ICU infection control link nurses, serving as the intermediary hub between the intensive care unit and the infection control team, juggling dual responsibilities, are challenging and require a variety of information and support.

**Implications for Nursing Management:** Hospital administrators should understand infection control link nurses' work experiences to implement targeted interventions promoting their work adaptation and motivation, thus reducing the incidence of healthcare-associated infections.

## 1. Introduction

Healthcare-associated infections (HAIs) represent a global public health issue [1]. They not only exacerbate patients' underlying conditions, prolong hospital stays, and increase treatment costs but also lead to patient mortality, imposing both physical and psychological burdens on them [2]. According to statistics from the World Health Organization, 2.5%–14.8% of patients worldwide are affected by HAIs [2], with approximately 30% of these infections occurring in intensive care units (ICU) [3, 4].

Multiple studies indicate that 35%–55% of HAIs can be prevented through the implementation of infection control measures [5, 6], yet actual compliance rates for preventive measures remain at 45%–70% [7, 8]. A quasiexperimental study demonstrated that the infection control link nurse (ICLN) program significantly improved healthcare workers' compliance with preventive measures by 18.8% (*p* < 0.05) [9]. Furthermore, research by Ojanperä et al. corroborates the efficacy of ICLN interventions, showing a reduction in HAI incidence from 14.0% to 11.7% postintervention (*p* < 0.05) [10]. ICLNs serve as an intermediate hub between the clinical departments and the epidemiology department. Through direct patient contact, they promptly identify clinical infection issues and lead the dissemination and implementation of infection prevention measures [11]. A scoping review by Dekker et al. [12] reported that ICLNs play multiple roles in infection prevention and control: they serve as role models and advocates, enabling clinical frontline staff to learn and implement infection prevention and control practices, thereby promoting improvements in ward practices; they act as communicators by receiving education from the infection control team and transmitting the latest information to frontline staff; they function as supervisors to oversee infection prevention and control practices; additionally, they support audits and monitoring, aiding in the early detection of infection outbreaks.

Previous studies have investigated the role positioning [13], current status and influencing factors of work engagement [14], and role perception [15] of ICLNs, contributing to the development of this role to some extent. However, significant research gaps remain in the current literature: existing studies are confined to phenomenological descriptions and fail to explore the adaptive strategies used by ICLNs to address workplace challenges [15]. Furthermore, no research has systematically examined ICLNs' work experiences or their perceptions of clinical practice in ICUs with high nurse turnover rates [16]. Hence, this study specifically focuses on the experiences, barriers, and unmet professional needs of ICU ICLNs working in high-nurse-turnover clinical environments. The findings may assist hospital administrators in developing tailored interventions to improve job motivation among ICU ICLNs, reduce HAI incidence, and ultimately enhance nursing care quality.

## 2. Materials and Methods

### 2.1. Study Design

This study employed phenomenological research methods to investigate the work experiences of ICLN in general ICUs across 12 hospitals in Chongqing, China. Phenomenological methods constitute a qualitative research approach aimed at understanding and exploring individuals' experiences and feelings related to a common phenomenon from a first-person perspective [17]. This paper adheres to the Standards for Reporting Qualitative Research [18] and collects data through individual semistructured interviews, with data analysis conducted using Colaizzi's 7-step analysis method [19].

### 2.2. Setting and Participants

The study invited ICLNs working in general ICUs in Chongqing, China, to participate. Inclusion criteria encompassed possessing a nurse practitioner certificate, working as ICLNs in general ICUs, having at least 2 years of work experience in the ICU, and being willing to participate in the study. Exclusion criteria included those who discontinued the interview for any reason. Participants were selected using purposeful sampling to maximize variation across different hospital grades, age groups, professional titles, and duration of work experience [20]. Researchers explained the research methods and objectives to the participants, and nurses who agreed to participate were invited to take part in individual semistructured interviews. The time and location of the interviews were determined and agreed upon beforehand by the researchers and participants. The interviews took place in a room at the participants' homes where they felt comfortable and alone.

### 2.3. Data Collection

The interview outline was developed based on a comprehensive literature review and consultations with nursing administrators and infection control professionals. After conducting two pilot interviews (which were included in the study data accordingly), no modifications were made to the interview guide. Open-ended questions explored the work experiences of ICLNs. The interview outline is presented in Table 1. The interviews were conducted between July and September 2024, using individual semistructured interviews to collect data. To accommodate the busy schedules of the nurses, participants were offered the choice of either face-to-face or online video interviews [21]. Specific interview times were notified to participants via WeChat beforehand. All participants provided informed consent and voluntarily participated in the study. The interviewer, who is the first author of this study, is a graduate student in nursing with a focus on nosocomial infections and has extensive experience in qualitative research, demonstrating proficient language skills and rich research expertise. The entire interview process was audio-recorded while ensuring the confidentiality of the participants' personal information. Before each interview, the interviewer introduced themselves and explained the research objectives to the participants to establish a rapport with all participants. At the beginning of the interview, a basic information survey was conducted to gain an in-depth understanding of participants' backgrounds and foster mutual trust. This was followed by open, exploratory, and in-depth questions focused on the research phenomenon. During the interviews, key points were taken while also documenting the participants' nonverbal behaviors, such as tone of voice, facial expressions, and body gestures. Data collection and analysis occurred concurrently. Data saturation was reached after the 10th participant, meaning that no new themes emerged at this point. This was further confirmed through two additional interviews [22]. The duration of the interviews ranged from 20 to 45 min, with an average interview time of 32 min.

### 2.4. Data Analysis

To minimize memory bias, audio-recordings were transcribed and coded within 24 h after each interview, and the transcripts and coded themes were returned to the participants for review to ensure factual accuracy. Participant information was kept confidential, with names anonymized using numerical codes. Two systematically trained researchers employed Colaizzi's 7-step analytical method [19] for data extraction and analysis, which includes becoming familiar with the data, identifying significant statements, constructing meaning units, clustering into themes, detailing the themes, generating a basic structure, and verifying the basic structure.

### 2.5. Validity and Rigor

The reliability of this study is ensured by adhering to standards of credibility, transferability, and confirmability [23]. Four members of the research team hold certificates of qualification in qualitative research training. The interviewer possesses deep theoretical insight, extensive scientific research experience, and adheres to strict data collection protocols. The adoption of phenomenological approaches throughout the research process ensures no manipulation or intervention with the research subjects, preventing personal value biases in understanding the phenomena. All researchers actively participated in various aspects of the research process. Additionally, this study refers to the Standards for Reporting Qualitative Research framework [18] to ensure credibility. Transferability is ensured by inviting participants from different institutions with diverse work experiences. Participant recruitment, interviews, and data analysis were conducted simultaneously to help researchers determine data saturation [22]. No repeated interviews were conducted. The data analysis process was conducted objectively, and the research results are based on the personal statements of the interviewees. The final themes and descriptions were verified by the interviewees themselves, accurately reflecting their experiences and demonstrating confirmability.

### 2.6. Human Ethics and Consent to Participate Declarations

This study has been reviewed and approved by the Hospital Ethics Committee (Approval No. [144] of Kelun in 2023) and complies with the Helsinki Declaration [24]. All participants voluntarily participated and provided informed consent, with the option to withdraw at any time. The privacy of participants was prioritized, and real names were replaced with numerical serial numbers.

## 3. Results

A total of 12 ICLNs from general ICUs were interviewed, including 4 from second-tier hospitals and 8 from third-tier hospitals. The average age of the participants was 36.5 ± 5.9 years, and 91.7% of the participants (*n* = 11) held a bachelor's degree. The duration of their work as ICU ICLNs ranged from 1 to 12 years. An overview of the basic information of the participants is presented in Table 2.

**Figure 1:**
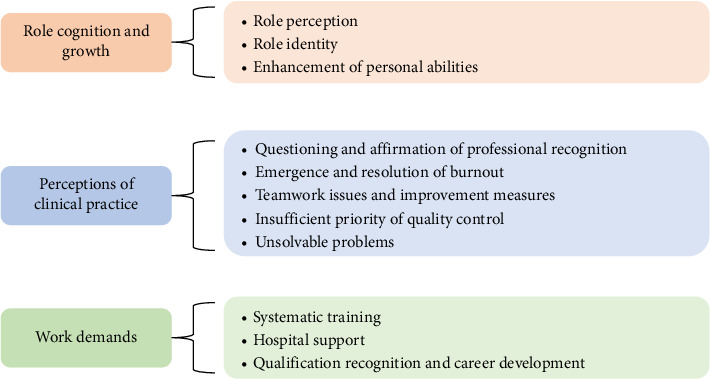
Three main themes and associated subthemes.

The interview data provided rich details that systematically revealed the growth trajectories, practical challenges, coping strategies, and evolving demands of ICU ICLNs during their professional tenure. Through the analysis of interview data, we identified 11 subthemes, which were categorized into three broad categories (as visualized in Figure 1): role cognition and growth (role perception, role identity, and enhancement of personal abilities); perceptions of clinical practice (questioning and affirmation of professional recognition, emergence and resolution of burnout, teamwork issues and improvement measures, insufficient priority of quality control, and unsolvable problems); and work demands (systematic training, hospital support, and qualification recognition and career development).

### 3.1. Role Cognition and Growth

#### 3.1.1. Role Perception

Functioning as a “middle hub,” the hospital ICLN plays a crucial role in directing, supervising, guiding, and educating clinical staff. Their responsibilities encompass overseeing infection prevention measures, implementing and monitoring evidence-based best practices, coordinating continuous education and training programs, conducting epidemiological investigations, and reporting infection data. They play a vital part in the prevention and control of hospital infections.

“ In essence, our primary responsibilities revolve around preparing for monthly surprise inspections, ensuring the completion of scoring criteria, compiling and reporting on ‘three-tube' data, and providing raw data to the epidemiology department. Furthermore, we integrate hospital infection control into our clinical work, striving not only to excel in our own practices but also to inspire and guide others to do the same. We offer correct instructions to those involved, thereby promoting quality improvement in hospital infection control.” (Nurse 11)

“As we (ICU infection control link nurses) are firmly rooted in the clinical frontline, we have the most direct and realistic view of the clinical situation, making our role exceptionally crucial (with a determined look). Furthermore, the effectiveness of infection prevention and control efforts primarily hinges on the frontline healthcare workers at the grassroots level. Our task is to provide timely reminders and oversight at every stage, striving to eliminate any oversights in these processes.” (Nurse 7)

#### 3.1.2. Role Identity

With the deepening understanding and implementation of hospital infection prevention and control work by ICU ICLNs, their enthusiasm and sense of responsibility toward this field have gradually increased. This is particularly evident when they witness improvements in patient outcomes, receive praise from patients' families, and gain trust and recognition from leaders and colleagues, thereby increasingly recognizing and appreciating their own value.

“Previously, I viewed myself solely as a nurse, believing that my role was confined to fulfilling my assigned duties, unrelated to hospital infection control. However, now (after taking on the role of an ICU infection control link nurse), I recognize that nurses play an indispensable part in hospital infection control, with significant responsibilities. Moreover, hospital infection control is not a simple matter (furrowing her brow); it requires constant contemplation of related issues and ongoing improvements.” (Nurse 1)

“Previously, I felt that nurses' roles were limited to giving injections and administering infusions (bitter smile). Nowadays, nurses must not only be proficient in giving injections and infusions but also understand ward management. We need to learn to consider all aspects, pay attention to patients' treatment quality and condition monitoring, and do our utmost!” (Nurse 2)

#### 3.1.3. Enhancement of Personal Abilities

As the infection control work of “top-down and bottom-up communication” has progressed, the ICU ICLNs have significantly enhanced their abilities to manage upward–downward communication and relationships. Moreover, as the number of issues encountered by these nurses increases, they are motivated to continuously learn and improve, leading to a gradual expansion of their professional knowledge and insights into infection control. This growth greatly enhances their professional competencies.

“In the past, all I needed to do was follow the hospital infection control protocols! But as my involvement in infection control work has deepened, I've gradually started to view issues from multiple angles, thinking more comprehensively and understanding more deeply. Especially with the enhancement of my upward and downward communication abilities, as well as my ability to handle relationships, the trust among colleagues has increased, and I've become more confident and responsible (smiling).” (Nurse 9)

“I'm quite familiar with the relevant systems at the national, hospital, and departmental levels. I can provide assistance to those in need, such as offering guidance on occupational exposure or prevention and control measures for patients with special infections. I'm also well-versed in the ideas and key points for preparing for inspections.” (Nurse 11)

### 3.2. Perceptions of Clinical Practice

#### 3.2.1. Questioning and Affirmation of Professional Recognition

Newly appointed ICLNs inevitably experience self-doubt and negation. Additionally, when overseeing and needing to correct the hospital infection prevention and control practices of frontline clinical staff, they often feel embarrassed and may also face suspicions regarding their professional competence. The support and trust from leaders, coupled with the enhancement of their own professional skills, are crucial in boosting their professional recognition.

“Initially, I lacked confidence and felt embarrassed to point out others' mistakes, fearing questioning. However, my leader encouraged me to trust in what I believed was right. Based on the support and trust from my leader, my current approach to overcoming embarrassment is to immediately point out and correct issues. Of course, this can be done either through gentle persuasion or mandatory enforcement, but it must be applied equally to everyone.” (Nurse 2)

“I think there are several things you can do to improve your professional recognition. First, keep a serious attitude. Second, be good at what you do and improve your communication skills. Third, fully grasp the knowledge related to infection control, such as relevant guidelines and clinical practice, and familiarize yourself with the work. Fourth, fully participate in infection prevention and control work, in the process of clinical practice, find and solve problems, recognize their own shortcomings and improve. Fifth, lead by example, promote the participation of all staff in quality control, correct each other's progress, and prove themselves with results. Sixth, always reflect and summarize, give timely feedback when there are problems, and seek advice from the head nurse, director, or hospital infection control department when unable to resolve them…” (Nurse 10)

#### 3.2.2. Emergence and Resolution of Burnout

ICU ICLNs are frontline clinical personnel and members of the infection control team. While fulfilling their clinical duties, they must also attend to tasks related to hospital infection management. When confronted with high stress, a severe blow to their confidence, or feelings of powerlessness, these nurses are prone to burnout. However, their persistence is fueled by a dual drive of maintaining a good mindset and a strong sense of responsibility.

“Burnout occurs when the tasks of statistical analysis pile up, when we have to meet various inspections and are blamed for any problems, when the end of the work on infection control is not as good as we would like it to be, when we are questioned for not doing a good job, and when we do not have as good a group cooperation as we would like it to be, and so on. For instance, analyzing infection cases can be manageable when there are no specific requirements for doctors but becomes difficult when there are. Delays are common… Additionally, when organizing departmental infection case analyses, participation is often low, and I often end up doing it alone (sigh), which frequently leads to feelings of helplessness.” (Nurse 9)

“Although I sometimes feel burnout, this is my responsibility, and I must do it. I just need to get through this period. During this time, I usually go out for dinners with friends to relieve my emotions, communicate, and take appropriate breaks to unwind. After that, I reflect on the motivations that keep me going, such as praise from my superiors, the significant improvements in the infection control work of our department, and the realization of my own sense of worth.” (Nurse 4)

#### 3.2.3. Teamwork Issues and Improvement Measures

“Everyone is a hospital infection control participant” serves as the cornerstone of infection prevention and control within departments, encompassing personal self-discipline, healthcare teamwork, and departmental infection control culture. Interviews have revealed that, while there are currently numerous challenges in team collaboration, these can be addressed through continuous oversight by the ICLN, mutual communication, and tolerance, as well as the establishment of appropriate reward and punishment systems.

“People differ from one another. Some individuals have yet to recognize the dangers of nosocomial infections and lack a sense of achievement in infection prevention and control. They also fail to set high standards for themselves, adhering to the mindset of doing the bare minimum and avoiding any extra effort. This results in a lack of personal discipline… However, the continuous supervision and emphasis by the infection control link nurse can, to a certain extent, improve the self-discipline of clinical frontline healthcare workers.” (Nurse 6)

“The members of our hospital infection control team are selected based on qualities such as meticulousness, encompassing nurses and doctors from various levels. This diversity in healthcare personnel allows for a broader and more diverse perspective on issues. Naturally, this also leads to potential conflicts, such as differing opinions between doctors and nurses. When such disagreements arise, we often resort to communication and coordination to reach a consensus. Additionally, we establish a relatively reasonable process to prevent similar issues from recurring in the future.” (Nurse 7)

“Infection control link nurses can take the lead in motivating department staff to implement infection prevention and control measures. However, the department's prevention and control culture remains weak (frowning). The reasons may include the high cost of infection prevention and control, less obvious benefits, insufficient attention across different levels… Therefore, there is an urgent need to cultivate a departmental culture where everyone is a hospital infection prevention and control ambassador. For instance, establishing a reasonable reward and punishment system for infection control work is crucial. Previously, our hospital would remind and rectify those who performed poorly, issue notifications in work groups, deduct performance bonuses, or impose fines, but the effects were not satisfactory. Currently, we require underperforming staff to participate in infection control tasks until replaced by the next individual. This new method has yielded promising results so far. Of course, those who perform well are rewarded with a bonus of 200 yuan (smiling).” (Nurse 5)

#### 3.2.4. Insufficient Priority of Quality Control

Hospital infection prevention and control should be an ongoing effort, with no room for negligence in this area. However, human energy is limited, and when ICLNs are under significant work pressure, priority must be given to essential clinical tasks. Furthermore, in the context of patient conditions, hospital infection prevention and control often takes a back seat. This is mainly manifested in healthcare providers' weaker awareness of prevention and control during emergency treatments, indicating a lack of emergency prevention and control consciousness.

“I am just a frontline clinical nurse, not a dedicated hospital infection control professional. You must prioritize and complete your primary duties before you can have the energy to focus on other tasks, such as hospital infection prevention and control.” (Nurse 11)

“For instance, when we arrive, we just perform hand hygiene quickly and proceed with necessary procedures, like intubating the patient if needed or catheterizing them for urination… Secondly, during emergencies, it's impossible to meet the 15-second requirement for wiping and disinfecting surfaces; we just dab the swab briefly before they need to administer medication. Prioritizing resuscitation efforts is unavoidable, and sometimes there isn't even enough time to take protective measures, including proper personal protective equipment for ourselves.” (Nurse 3)

#### 3.2.5. Unsolvable Problems

Currently, clinical settings face intractable problems such as a lack of hardware, insufficient manpower, and heavy workloads. These challenges significantly hinder healthcare providers from implementing effective hospital infection prevention and control measures.

“Some problems, once identified, may not necessarily be resolvable. For instance, inadequate handwashing facilities. Our department recently switched to a different brand of hand sanitizer that cannot be placed in the designated holder, so it has been moved from hanging at the end of the bed to being placed on the bedside table, which is quite inconvenient and has significantly reduced its usage.” (Nurse 12)

“Asking me to handle both clinical duties and hospital infection control is a huge strain on my time and energy (sigh). I believe that ICUs, as departments with high personnel turnover, are particularly prone to increased hospital infection rates due to inadequate infection control measures if management oversight or attention is lacking. Therefore, there is a pressing need for infection control nurses. Of course, I understand that this requires manpower, and the issue of human resources is a major challenge.” (Nurse 8)

### 3.3. Work Demands

#### 3.3.1. Systematic Training

Most of the interviewees became the ICU ICLNs due to the recommendation of their head nurses. However, the selection criteria varied among different head nurses, leading to a discrepancy in the professional competence of ICU ICLNs across hospitals. The interviewees believed that both new and experienced ICLNs required systematic and professional training, enabling professionals to do professional work. They also suggested exchanging ideas and learning from hospitals or departments that performed better in this area, aiming for mutual improvement.

“Although we are infection control link nurses, we haven't actually participated in any systematic training. Our learning has been scattered and piecemeal, such as attending occasional lectures held by the epidemiology department. We need systematic and professional training to guide us on how to do and implement things! We also need to continuously learn the latest infection control knowledge and make progress to update and improve the existing systems. Meanwhile, we should go out and learn from others' practices that are worthwhile, such as their superior infection control concepts and management (gesturing with hands). Additionally, when borrowing from others' experiences, we need to localize them because certain measures may not be universally applicable due to differences in regional layouts and other factors.” (Nurse 4)

“Regulations and policies are the prerequisites, but the key lies in how to implement them? How to carry out inspection work? How to excel in infection control? And from which aspects should we proceed? Currently, the existing infection control guidelines and industry standards are outdated and lack updates, while the updated content is too general and inconvenient for clinical implementation… Infection control link nurses often promote the implementation of measures in clinical settings based on their own understanding of guidelines and norms. However, such understanding varies among individuals, which may lead to certain misunderstandings or blind spots. Therefore, external training and learning are crucial. By exchanging experiences, we can collect relevant knowledge and solve blind spots that we ourselves cannot address.” (Nurse 8)

#### 3.3.2. Hospital Support

Hospital support and reasonable institutional support are the guarantees for motivating the work enthusiasm of ICU ICLNs. The degree of importance attached by the hospital and the subsequent institutional or policy support, to a certain extent, influence the effectiveness of hospital infection control efforts.

“I hope the hospital can establish a reasonable management system for infection control link nurses. This system should provide scheduling support and performance incentives, allocate more time for infection control tasks, and offer certain compensation—such as financial rewards, certificates, or honors (smiling). Further, strong support should be given to the qualification training of infection control link nurses by providing systematic training, opportunities for external learning, or participation in conferences.” (Nurse 6)

“Abolish some unnecessary punishment systems. For example, when it comes to microbiological monitoring results, if you directly deduct points or money for issues found, I find it very confusing. The purpose of conducting this inspection is to identify problems, but instead of addressing them, you penalize those who find them. To avoid penalties, people may resort to falsification, which I think is completely unnecessary (annoyed)!” (Nurse 11)

#### 3.3.3. Qualification Recognition and Career Development

Currently, the qualification training and accreditation system for ICLNs in China are still at an early stage of development. Additionally, there is a lack of structured career development planning for this role. Respondents expressed their desire for relevant and authoritative qualification recognition, as well as the existence of a clear career development path.

“In the future, I hope that infection control link nurses will require qualification recognition. For instance, they should possess 2–3 years of clinical-related prevention and control experience and undergo systematic training and assessment before taking on the role.” (Nurse 5)

“ I hope the qualification certificate for hospital infection control link nurses will have high professional value. For example, after obtaining this certificate, we can compete for management positions in hospital infection control departments or clinical units across the entire city or country. In terms of career development, they can serve as local or regional experts in hospital infection control to provide corresponding guidance or engage in mutual learning and exchange.” (Nurse 9)

## 4. Discussion

In this study, qualitative interviews revealed the work experiences of ICU ICLNs, including role cognition and growth, perceptions of clinical practice, and work demands. Serving as an “intermediary hub” between the clinical departments and the epidemiology department, ICLNs demonstrated continuous professional growth and gradually developed role identity. Participants unanimously agreed that dealing with problems in practice was essential for accumulating practical experience. Additionally, they emphasized the necessity of hospital support to facilitate continuous learning and experience exchange. Globally, training models for ICLNs vary significantly [25–27]. Compared to countries with mature ICLN systems such as the United Kingdom and the United States, China's ICLN development currently relies heavily on experiential learning, lacking a standardized qualification training and certification framework [28–30]. To address this, we recommend integrating international best practices with the characteristics of China's healthcare system to establish a localized professional training system for ICLNs.

Through qualitative interviews, this study reveals that the role recognition and growth experienced by nurses after becoming ICU ICLNs contribute to their self-positioning and work motivation. Previous research has primarily focused on the functional responsibilities and role positioning of ICLNs, with less attention given to the reasons for their role identity and personal growth within the role [13–15]. Notably, emphasizing ICLNs' role cognition and professional growth fosters a positive cycle of role identity and professional development. This cycle facilitates their transition from “passive implementers” to “proactive leaders in infection control.” The study reveals that as ICLNs become more involved in hospital infection prevention and control efforts, their understanding and identification with their roles gradually deepen. This may be attributed to the current poor adherence of healthcare staff to recommended infection prevention and control practices, while ICLN programs improve clinical staff compliance with infection control measures [8, 9, 26, 31]. It also relates to the ongoing need in high-turnover ICUs for ICLNs to educate and support less experienced nurses [16]. Additionally, this connects with the demonstrated ability of ICLN programs to reduce HAI incidence, HAI-related deaths, and hospital stays [19, 26, 32]. Our research further indicates that the enhancement of personal abilities contributes to individuals realizing their own value and increasing their job motivation. Serving as an ICLN not only enhances their communication skills, relationship management skills, and problem-solving abilities but also increases their professional competence and unique insights, significantly boosting their self-confidence and work capabilities. Therefore, hospital administrators should prioritize understanding ICLNs' role identity and professional growth to enhance the effectiveness of ICLN programs. Future studies are warranted to explore targeted intervention strategies and establish sustainable mechanisms for fostering ICLNs' role identity.

Unlike previous studies [13–15], this research focuses on the perceptions of ICU ICLNs toward clinical practice. It not only uncovers the difficulties faced by ICU ICLNs in their clinical work but also summarizes their strategies to overcome these challenges. This provides valuable insights and recommendations for ICU ICLNs, particularly those who are newly inducted, thereby facilitating their work adaptability to a certain extent. For instance, newly appointed ICLNs may encounter embarrassment or skepticism when supervising and guiding others, necessitating an enhancement of their professional recognition. The participants in this study indicated that support and trust from leaders, the improvement of self-professional competence, and self-confidence are crucial for enhancing professional recognition. Currently, there is a lack of clarity regarding the job satisfaction and burnout among ICLNs [33, 34]. However, according to the participants in this study, burnout often arises in specific contexts. These contexts include a pile-up of data analysis tasks, the need to prepare for various inspections while facing blame for issues, dissatisfaction with the outcomes of hospital infection prevention and control efforts, being questioned about their performance, and poor team collaboration. According to the study results, burnout among frontline clinical staff can be mitigated through continuous supervision and guidance by ICLNs. For ICLNs themselves, addressing their work-related burnout requires the dual drive of maintaining a good mindset and a sense of responsibility. A positive departmental prevention and control atmosphere and effective team collaboration play significant roles in enhancing the work enthusiasm of ICLNs [16, 35]. Therefore, it is crucial to regulate personal self-discipline, promote doctor–nurse collaboration through communication, and establish a prevention and control culture where “everyone is a hospital infection control officer.” Of course, there are also some intractable difficulties, such as poor infection control awareness during emergencies, lack of hardware and manpower, and heavy workloads. Currently, there is a lack of solutions in this area [36], and future research can further explore these issues.

Previous research distributed 1938 questionnaires to investigate the job demands of ICLNs, and the results revealed that the current systematic training system for ICLNs needs further optimization [13]. However, this study further delves into the work demands of ICU ICLNs through qualitative interviews, providing supplementary insights into their work demands to a certain extent. Our research emphasizes the need for hospital support system training and external exchange and learning opportunities for ICLNs. According to the findings, systematic training is required to continuously acquire the latest infection control knowledge, thereby updating and improving existing protocols. Additionally, collecting relevant experiences through external training can address blind spots that ICLNs themselves are unable to resolve. These research results are similar to those of current studies [26, 35, 37]. Dekker et al. [35] reported that systematic training and education for ICLNs are considered core elements of ICLN programs, suggesting a multimodal approach to their education to prevent job vulnerability. For instance, Lee and Yang [37] proposed scenario-based infection control simulation training. Furthermore, Peter et al. [26] indicated that, in addition to technical knowledge training, psychological skills training is crucial for ICLNs. Currently, the qualification training and recognition of ICLNs in China are still in their preliminary stages of development [30]. With the pursuit of value by ICLNs, they hope for rigorous qualification training and clear career development pathways in the future, further promoting their work enthusiasm and value realization.

### 4.1. Limitation

The present study has several limitations. In qualitative research, concepts are interpreted within specific contexts and cultures, posing challenges to the generalization and application of this study's findings in other settings. Remote online meetings may limit the view of participants' body language. Furthermore, technical issues such as network disruptions during online meetings can occur, affecting the continuity and fluidity of the interviews to some extent. Additionally, the lack of integration with quantitative research in this study hinders the identification of systematic training content. Future research should adopt a mixed-methods approach combining both qualitative and quantitative research based on an expanded sample selection range to provide more convincing and precise insights.

## 5. Conclusion

This study is recognized as the first to introduce the work experiences of ICU ICLNs. For newly recruited ICU ICLNs, these experiences are particularly invaluable. According to the findings of this study, the work experiences of ICU ICLNs mainly encompass three components: role cognition and growth, perceptions of clinical practice, and work demands. The results of this study may facilitate the work adaptation of ICU ICLNs and prevent job vulnerability. Meanwhile, it contributes to promoting further refinement of systematic training, credential recognition, and career development planning for ICLNs in China, aiming to create a supportive work environment. Therefore, it is recommended that hospital administrators understand the work experiences of ICU ICLNs, formulate targeted intervention measures, maximize their clinical effectiveness, enhance the implementation rate of infection prevention and control measures among clinical healthcare workers, and ultimately reduce the incidence of nosocomial infections.

## Figures and Tables

**Table 1:** Semistructured questionnaire.

	Questions
1	Please describe specifically what infection prevention and control work you are involved in. What is your role?
2	Do you feel that your current job fits your understanding of what an infection control link nurse is?
3	How has becoming an infection control link nurse affected you?
4	What difficulties have you encountered in carrying out your work since you became the ICU infection control link nurse? How were they handled? What help have you received? What help would you like to receive?
5	What do you think are the barriers and facilitators to the implementation of hospital infection prevention and control measures by frontline clinical staff?
6	What are your expectations and needs for ICU infection control link nurse qualification training?

**Table 2:** Demographic information for the infection control link nurses (*n* = 12).

Characteristics	Categories	Nurses, *n* (%)
Age (years)	Mean ± SD (range)	36.5 ± 5.9 (26–47)
	26–35	6 (50.0%)
	36–40	2 (16.7%)
	≥ 41	4 (33.3%)

Gender	Female	11 (91.7%)
	Male	1 (8.3%)

Professional title	Junior professional title	2 (16.7%)
	Intermediate professional title	9 (75.0%)
	Senior professional title	1 (8.3%)

Education level	College degree	1 (8.3%)
	Bachelor's degree	11 (91.7%)

Hospital grade	Second-tier Grade A	4 (33.3%)
	Third-tier Grade A	8 (66.7%)

Years of work experience	Mean ± SD (range)	15.3 ± 6.6 (3–26)
	≤ 10	2 (16.7%)
	11–20	7 (58.3%)
	> 20	3 (25.0%)

Years as ICU infection control link nurses	Mean ± SD (range)	5.6 ± 3.6 (1–12)
	≤ 5	7 (58.3%)
	6–10	3 (25.0%)
	> 10	2 (16.7%)

## Data Availability

The data used to support the findings of this study are available from the corresponding author upon reasonable request.
